# Literary Notes

**Published:** 1903-01

**Authors:** 


### LITERARY NOTES.

MESSRS. P. BLAKISTON'S SON & CO. are publishing "Biographical Clinics; the origin of the ill-health of DeQuincey, Carlyle, Darwin, Huxley and Browning," by Dr. George M .Gould. The author has gathered from the biographies, writings and letters of the five named men every reference to their ill-health. Each endured, as is well known, a life of suffering which made almost every day a torment and by which their work and worth as an asset of the nation and civilisation was conditioned and often rendered morbid. The cause of their affliction was an utter mystery to their physicians. Dr. Gould's conclusion from logic and from a careful summary of the clinical symptoms, is that each of the writers suffered from eyestrain, and that scientific correction of their ametropia would have transformed their lives of mystery into lives of happiness. A history of the discovery of astigmatism and eyestrain, with a discussion of its indications and responsibilities, completes the work. It is interestingly written, and will undoubtedly meet with a ready sale among medical men and those interested in the works and lives of these great writers.

---

THE *New York Medical Journal* in its issue of December 20, 1902, publishes a report of the clinic of Professor Lorenz, held at the hospital for the ruptured and crippled, his first demonstration in New York, illustrated with an accurate half-tone engraving.

